# Probiotic cell-free supernatants as a strategy against antimicrobial resistance: a systematic review

**DOI:** 10.3389/fcimb.2026.1731341

**Published:** 2026-03-02

**Authors:** Maisah Meyhr D’Carmo Sodré, Ian David Araújo Cruz, Uener Ribeiro Santos, Sheila Cristina Potente Dutra Luquetti, Vânia Lúcia Silva, Alessandra Barbosa Ferreira Machado, Cláudio Galuppo Diniz, Cláudio Teodoro de Souza, Carla Cristina Romano, Lauro Juliano Marin, Luciana Debortoli de Carvalho

**Affiliations:** 1 Department of Biological Sciences, Microbiology Laboratory, Santa Cruz State University, Ilhéus, Bahia, Brazil; 2 Postgraduate Program in Biology and Biotechnology of Microorganisms, Microbiology Laboratory, Santa Cruz State University, Ilhéus, Bahia, Brazil; 3 Ages Faculty of Medicine of Irecê, Biomedicine Collegiate, Irecê, Bahia, Brazil; 4 Nutrition Faculty, Federal University of Juiz de Fora, Juiz de Fora, Minas Gerais, Brazil; 5 Institute of Biological Sciences at Federal University of Juiz de Fora, Juiz de Fora, Minas Gerais, Brazil; 6 Faculty of Medicine, Federal University of Juiz de Fora, Juiz de Fora, Minas Gerais, Brazil; 7 Health Department, Santa Cruz State University, Ilhéus, Bahia, Brazil

**Keywords:** alternative therapies, antimicrobial resistance, cell free supernatant, one health, systematic review

## Abstract

Antimicrobial resistance (AMR) is a critical global health threat that may cause up to 10 million deaths annually by 2050, requiring integrated actions within the One Health framework. The misuse of antimicrobials across human, animal, and environmental sectors has intensified the spread of multidrug-resistant bacteria, including *Escherichia coli, Staphylococcus aureus*, and *Klebsiella pneumoniae*. In this context, *Lactobacillus*-derived postbiotics have emerged as eco-friendly alternatives with antimicrobial and antibiofilm properties. A systematic review was conducted to consolidate scientific evidence on the strategic potential of Lactobacillus cell-free supernatants, with a specific focus on *Limosilactobacillus fermentum*. Studies published between 2000 and July 2025 were screened, prioritizing investigations that evaluated antimicrobial activity, biofilm inhibition, and efficacy in biological and technological models against multidrug-resistant pathogens. After screening, 95 studies were included in the analysis. *L. fermentum* was deliberately selected as the focus species based on consistent evidence of postbiotic efficacy against pathogenic bacteria and biofilm formation. The reviewed studies also demonstrated favorable physicochemical stability of *L. fermentum* cell-free derivatives, supporting their safety and scalability for applied use. This review highlights *L. fermentum* as a strategic model within One Health aligned approaches to combat AMR. The findings reinforce the role of postbiotics as sustainable, effective, and scalable tools for mitigating antimicrobial resistance across human, animal, and environmental interfaces.

## 1 Introduction

Antimicrobial resistance (AMR) has emerged as one of the most critical global health challenges, driven primarily by the inappropriate and excessive use of antibiotics across human, animal, and environmental sectors (Ahmad et al., 2021; Ghimpeţeanu et al., 2022). Clinically relevant multidrug-resistant (MDR) bacteria, such as *Escherichia coli*, *Staphylococcus aureus*, *Pseudomonas aeruginosa*, and *Klebsiella pneumoniae*, are frequently associated with resistance to β-lactams, carbapenems, and fluoroquinolones. These resistance patterns severely limit therapeutic options, increase morbidity and mortality, prolong hospitalization, and impose substantial economic burdens on healthcare systems (Mackenzie and Jeggo, 2019; Sinclair, 2019; World Health Organization, 2022; Salam et al., 2023). The World Health Organization estimates that AMR could cause up to 10 million deaths annually by 2050 if no effective interventions are implemented (Michael et al., 2014).

**Figure 1:**
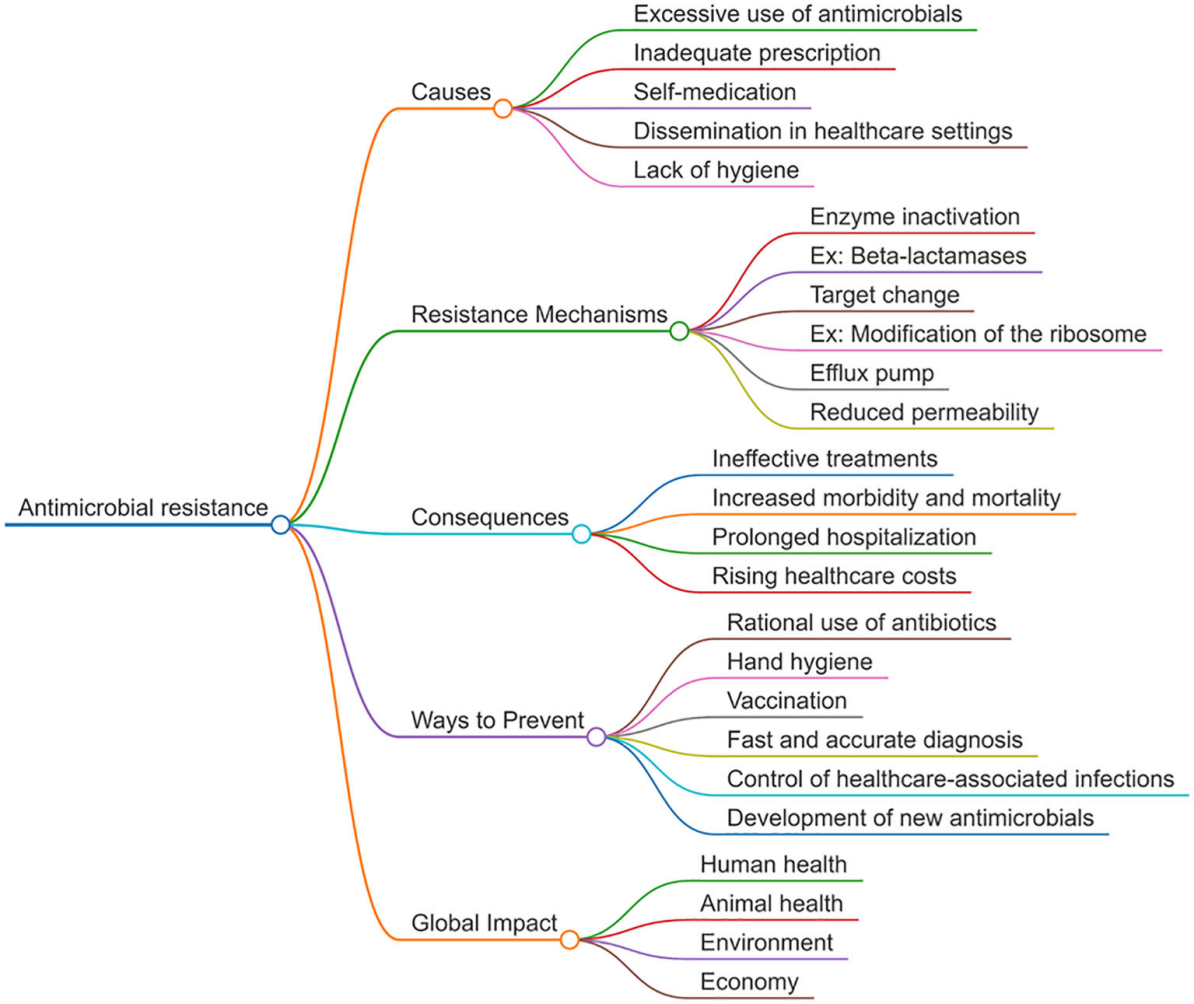
Antimicrobial Resistance: Causes, Mechanisms, and Consequences.

Microorganisms possess remarkable adaptability, enabling them to withstand selective pressures and disseminate resistance determinants through vertical and horizontal gene transfer (Pang et al., 2019). Antimicrobial resistance may be intrinsic, arising from inherent structural or functional traits, or acquired through mutations and horizontal gene transfer. Acquired mechanisms are generally classified into three main categories: (i) reducing intracellular antimicrobial concentrations through limited membrane permeability or efflux pumps; (ii) modifying antimicrobial targets via genetic mutations or post-translational modifications; and (iii) enzymatically inactivating antimicrobial agents through hydrolysis or chemical modification (Blair et al., 2014; Manyi-Loh et al., 2018; CDC, 2019). These multifactorial processes illustrate how antimicrobial resistance arises and spreads through interconnected biological and environmental pathways (**Figure 1**).

The *One Health* framework integrates all dimensions of health, from the identification of underlying causes to the assessment of their broader ecological and societal impacts. It acknowledges the intricate interconnections among human, animal, and environmental health, fostering coordinated and multisectoral collaboration (Obiebe et al., 2023; Ahmed et al., 2024; Kaur et al., 2024). Environmental reservoirs, such as pharmaceutical effluents, untreated wastewater, and agricultural run-off, further exacerbate this global threat. Consequently, effective mitigation of AMR requires policies and interventions that transcend clinical boundaries and incorporate comprehensive ecological perspectives (Nappier et al., 2020; Liguori et al., 2022).

**Figure 2:**
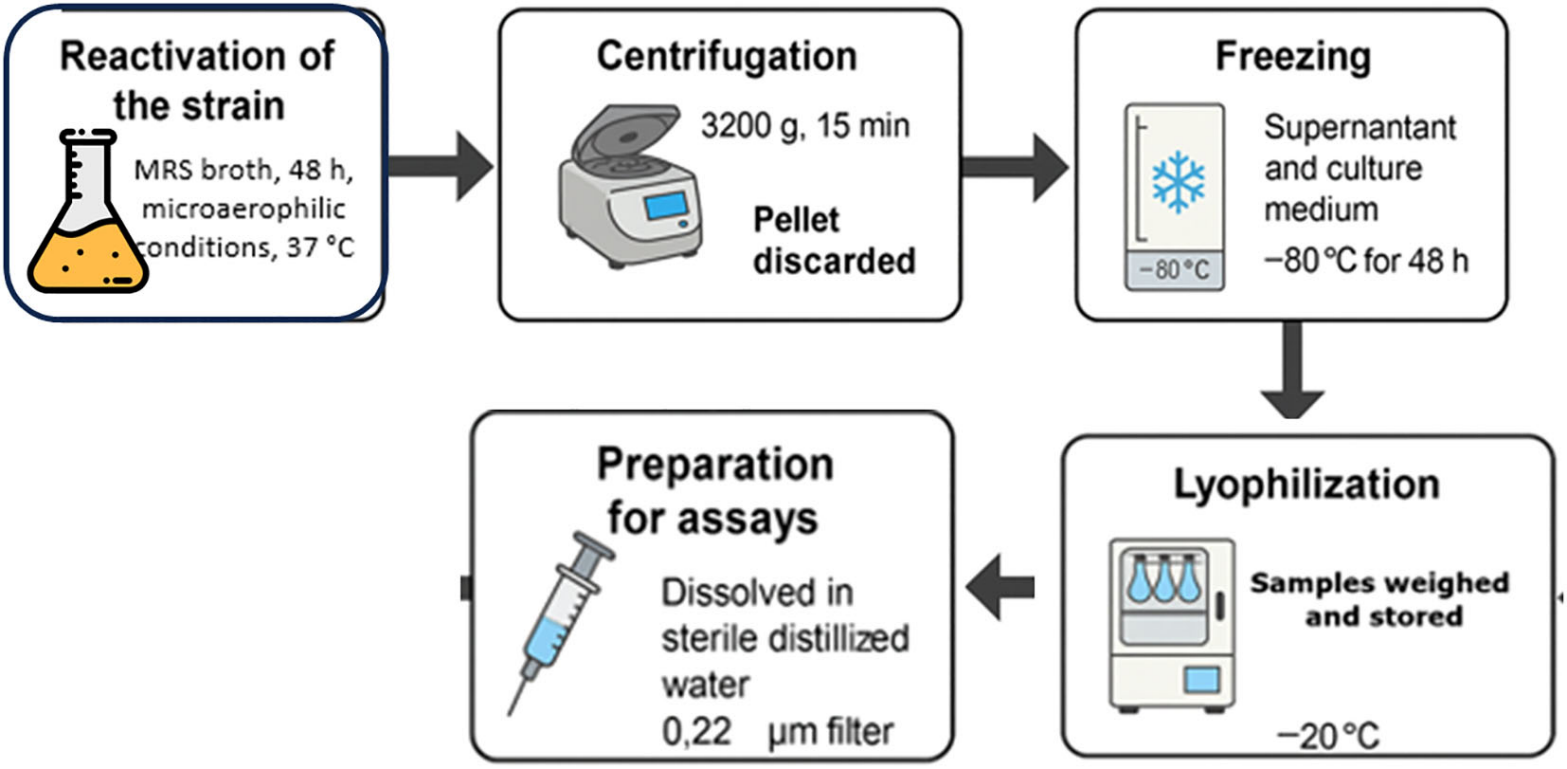
Schematic representation of the methodology used for the preparation of the bacterial cell-free supernatant.

In response to this urgent global threat, alternative strategies have gained momentum, particularly those exploring the potential of probiotics and their cell-free supernatants (CFS) as innovative tools for infection prevention and AMR mitigation (Cuevas-González et al., 2020; Abbasi et al., 2021; Warda et al., 2021). The CFS represents the extracellular fraction obtained after the removal of bacterial cells by centrifugation and filtration, containing metabolites such as organic acids, bacteriocins, biosurfactants, and peptides (Choi et al., 2022) (**Figure 2**). These compounds act through multiple mechanisms, interfering with microbial growth, disrupting biofilm formation, and modulating virulence, thus offering a safe, sustainable, and complementary approach to conventional antimicrobial therapy (Xu et al., 2022; Gholami et al., 2023; Zanetta et al., 2023).

Among probiotic species, *Lactobacillus* strains, including *L. acidophilus*, *L. casei*, and *L. rhamnosus*, have been widely applied in food and health formulations (Shah et al., 2024). Based on consistent evidence, *Limosilactobacillus fermentum* was deliberately selected as the focus species for this review. Its postbiotics exhibit multifunctional activity, notably inhibiting microbial colonization and biofilm development, while their physicochemical stability reinforces their potential for safe and scalable applications (Zhong et al., 2024).

This review synthesizes two decades of evidence and highlights postbiotics, particularly those derived from *L. fermentum*, as innovative, eco-friendly strategies aligned with the *One Health* approach to combat AMR. Although numerous *in vitro* and *vivo* studies have evaluated the antimicrobial activity of *L. fermentum* supernatants, the evidence remains fragmented, with a lack of comprehensive synthesis. To the best of our knowledge, no systematic review has yet addressed this topic. Therefore, this study aims to compile and critically assess current evidence on the use of CFS from *Limosilactobacillus fermentum* as a promising alternative strategy to tackle AMR, emphasizing its potential within the *One Health* perspective that integrates human, animal, and environmental health.

## 2 Materials and methods

### 2.1 Data sources and research strategy

This systematic review was conducted following the Preferred Reporting Items for Systematic Reviews and Meta-Analyses Protocols (PRISMA-P) guidelines (Moher et al., 2015). The research problem and guiding questions were defined and structured through the development of a protocol (**Supplementary Table SM1**). The literature search covered publications from 2000 to 2025 and was conducted in three electronic databases: PubMed, Scopus, and Science Direct. Preliminary searches were performed to refine the keyword set and construct the final search strings, which were adapted to the specific syntax of each database. The search strategy combined controlled vocabulary terms (MeSH/DeCS) and free-text keywords. The terms used included: “*Limosilactobacillus fermentum*” OR “*Lactobacillus fermentum*” OR “*L. fermentum*”, AND “cell-free supernatant” OR “culture supernatant” OR “fermentation supernatant” OR “CFS” OR “postbiotic”, AND “antimicrobial” OR “antibacterial” OR “antimicrobial activity” OR “antimicrobial effect” OR “pathogen inhibition”. These terms were selected based on the central theme of this review and the specific interest of our research group in evaluating the antimicrobial potential of cell-free supernatants derived from *Limosilactobacillus fermentum.*

### 2.2 Inclusion criteria and selection process

Initially, duplicate records were removed from all retrieved articles. Subsequently, studies were selected according to the PICOS framework: the population comprised pathogenic microorganisms (bacteria and/or fungi/virus) exposed to CFS of *Limosilactobacillus fermentum*; the intervention involved treatment with these CFS derived from *L. fermentum*; the comparator included studies with or without control groups, or those employing other supernatants for comparison; the outcomes encompassed antimicrobial activity, as assessed by microbial growth inhibition, inhibition zone diameter, minimum inhibitory concentration (MIC), or other relevant measures of efficacy; and the study designs included experimental studies conducted *in vitro* and *in vivo*, published in scientific journals without date restrictions, from 2000 to 2025. Only original research articles published in English or Portuguese were considered. Exclusion criteria comprised review articles, duplicated results, case reports, book chapters, and conference abstracts.

The study selection process consisted of two stages. In the first stage, titles and abstracts were screened according to the above criteria for preliminary inclusion. Articles that met the criteria at this stage underwent full-text assessment in the second stage, during which additional exclusions were applied based on the eligibility criteria. A local database was then established containing all articles that passed both stages of selection. Any uncertainties regarding study eligibility were resolved through discussion among the research team.

### 2.3 Quality assessment and risk of bias evaluation

The methodological quality and risk of bias (RoB) of all included studies were systematically assessed after the selection process. Considering the predominantly experimental and laboratory nature of the studies, a critical appraisal tool adapted from the Joanna Briggs Institute (JBI) checklist was used for quality assessment. Each study was independently assessed by two reviewers according to methodological domains that included clarity of objectives, adequacy of experimental design, standardization and transparency of postbiotic preparation protocols, reproducibility of antimicrobial and antibiofilm assays, and clarity in the analysis and reporting of data (Aromataris et al., 2015). Discrepancies in the assessments were resolved through discussion and consensus. This approach allowed for a qualitative stratification of the studies regarding their methodological quality, enabling a critical assessment of the overall strength and reliability of the evidence and guiding the interpretation of the results of the review.

## 3 Results

### 3.1 Search results and publication trends

**Figure 3:**
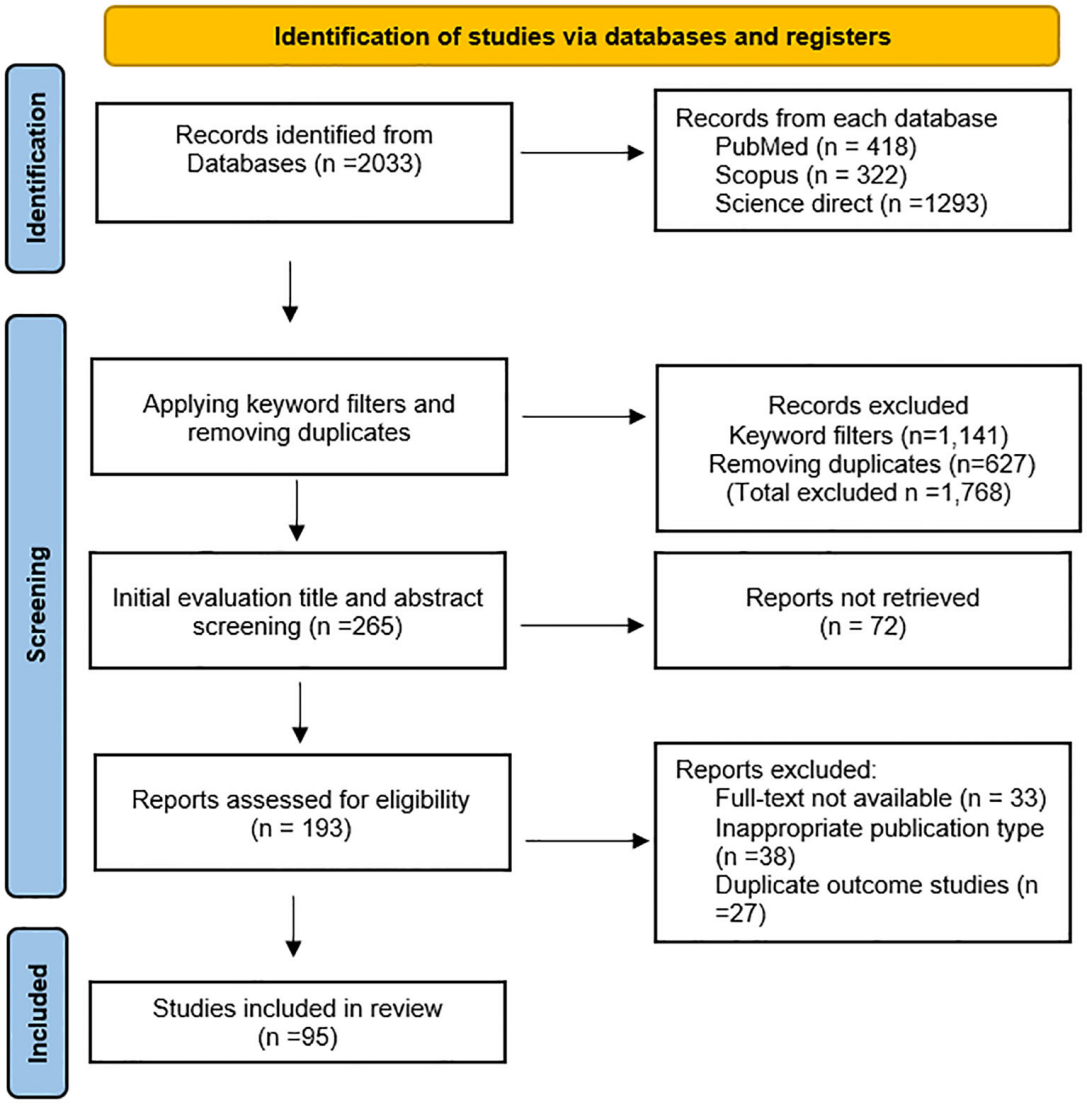
Schematic representation of the steps followed to compile the final database of this systematic review.

A total of 2,033 records were identified from three databases: PubMed (n = 418), Scopus (n = 322), and ScienceDirect (n = 1,293). After applying keyword filters and removing duplicates, 1,768 records were excluded. The remaining 265 records were screened by title and abstract, and 72 reports were not retrieved. Subsequently, 193 articles were assessed for eligibility. Among these, 33 studies were excluded due to unavailability of full text, 38 due to inappropriate publication type, and 27 due to duplicate outcomes. In total, 95 studies met the inclusion criteria and were included in this systematic review (**Figure 3**).

**Figure 4:**
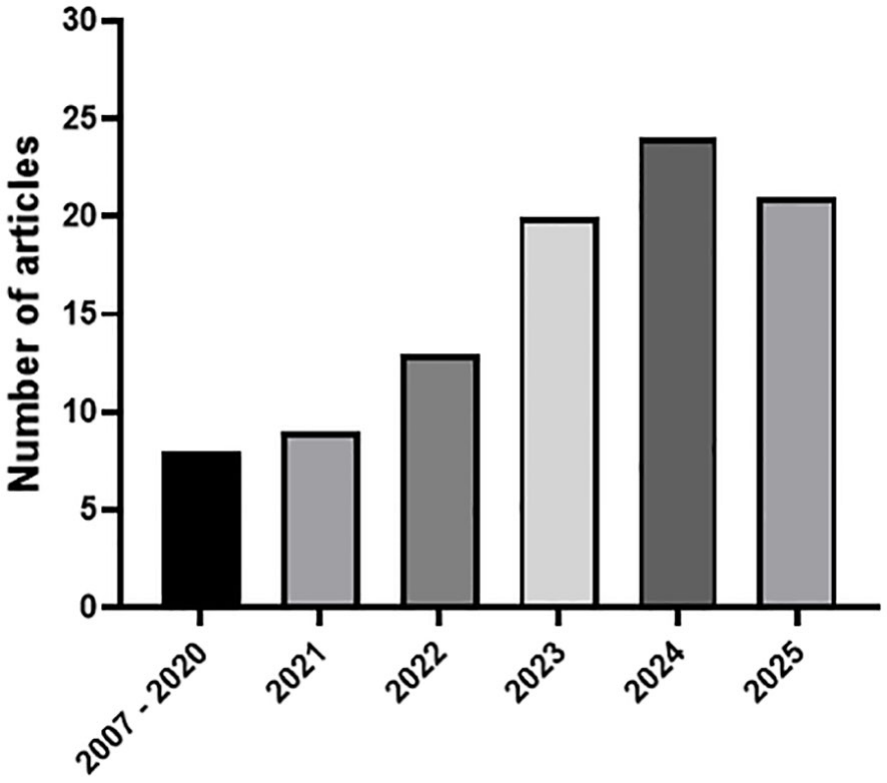
Time trend of publications.

Due to the low number of publications per year, particularly between the early 2000s until 2020, when in some years only one study met our inclusion criteria, we decided to group these years into broader periods. The temporal analysis revealed a progressive increase in the number of studies over time, with a more pronounced concentration between 2023 and the first half of 2025, reflecting the growing scientific interest and production in this area (**Figure 4**).

**Figure 5:**
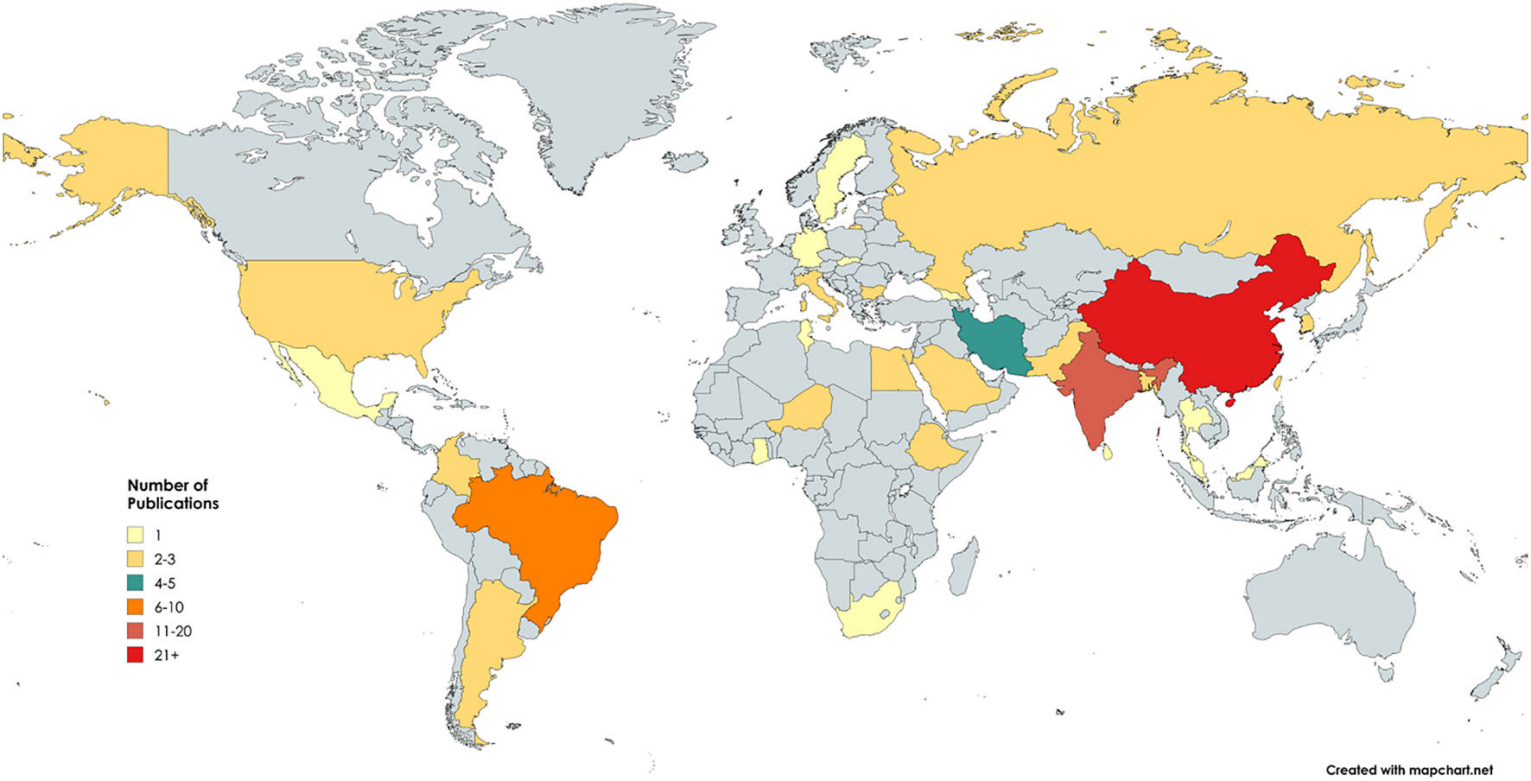
Geographical distribution of publications.

Analysis of the geographic distribution of the included studies revealed that research on this topic is conducted globally, although it is concentrated in specific countries. China accounted for the highest number of publications, with 26 studies (27.4%), followed by India with 12 studies (12.6%) and Brazil with 7 studies (7.4%). Other countries contributed smaller numbers of studies, ranging from 1 to 5 each. When grouped by continent, most publications originated from Asia, followed by the Americas, Europe, and Africa, reflecting the predominance of Asian research centers in this field. In total, 28 countries contributed to studies, underscoring both the global relevance of the topic and the geographic diversity of the samples analyzed (**Figure 5**).

### 3.2 Origin of sample isolation

Analysis of the included studies revealed that most samples originated from fermented dairy products (27 studies), such as cheeses and breads, reflecting the research interest in functionally active foods. Other fermented foods were also investigated, alongside commercial isolates (13 studies). Samples of human origin (14 studies) comprised isolates from women with bacterial vaginosis and/or candidiasis, human feces, breast milk, and oral samples, highlighting the attention given to the human microbiota and health. Additionally, the studies analyzed lactic acid bacteria (LAB) isolates obtained from fermented foods (12 studies) such as kimchi and pickles, fermented beverages (9 studies) such as wine and beer, and various samples grouped as “other” (11 studies). The latter included plant extracts, avocado pulp, dairy residues, soybean dairy residues, LAB isolates from apple varieties, isolates from the intestinal tract of buffalo and animal feces, as well as experimental models in mice fed a high-cholesterol diet.

Lower representation was observed for *Lactobacillus* spp. strains isolated from the intestinal tract of chickens (3 studies), LAB strains from *Theobroma cacao* fermentation (3 studies), and isolates from meat intended for human consumption (3 studies). Overall, these data demonstrate that research predominantly focuses on fermented foods and human-derived products, while other sources remain underexplored, revealing gaps that may guide future studies (**Table 1**).

**Table 1:** Origin of *Lactobacillus* spp. isolates included in the systematic review.

Sample source	Subcategory/examples	No. of studies
Fermented dairy products	Cheese and bread	27
Fermented foods	Pickled/processed vegetables	12
Fermented beverages	Wine and beer	9
Human origin	Women with bacterial vaginosis and/or candidiasis, feces, breast milk, oral samples	14
Commercial	—	13
Others	Plant extract, avocado pulp, dairy residues (milk and soy), animal feces, buffalo intestinal tract, apple-derived lactic acid bacteria, murine model with high-cholesterol diet	11
*Lactobacillus* strains from animals/plants	Chicken intestinal tract, *Theobroma cacao* fermentation	6 (3 + 3)
Meat intended for human consumption	—	3

### 3.3 Study designs and characteristics of included studies

Analysis of the included studies revealed that the majority employed *in vitro* approaches (60 studies), highlighting the predominance of controlled laboratory experiments. Studies combining *in vitro* with other methodologies (15 studies) were also frequent, whereas approaches such as *in vivo* (4 studies), *in situ*, metagenomics, and *in silico* were less represented.

The “Other” category (6 studies) encompassed advanced experimental methods and combinations of approaches, including genomics integrated with *in vitro* assays, studies using dairy products (e.g., yogurt), randomized, double-blind, placebo-controlled clinical trials, safety assessments combining in silico and *in vitro* tests, multi-omics laboratory investigations (metabolomics, transcriptomics, and proteomics), co-cultivation of LAB strains to enhance bacteriocin production, *in vitro* isolation and probiotic characterization of *Lactobacillus* spp., and combined *in vitro* and *in vivo* evaluations using animal models and *Caenorhabditis elegans*.

Regarding methodological quality, the risk of bias assessment indicated that most studies presented moderate methodological rigor, mainly due to heterogeneity in experimental protocols and partially description of critical parameters, such as standardization of controls and reproducibility of assays. A smaller proportion of studies demonstrated high methodological quality, typically associated with more structured experimental designs and comprehensive reporting practices, involving applications ranging from *in vitro* activities to well-defined bioinformatics approaches.

**Figure 6:**
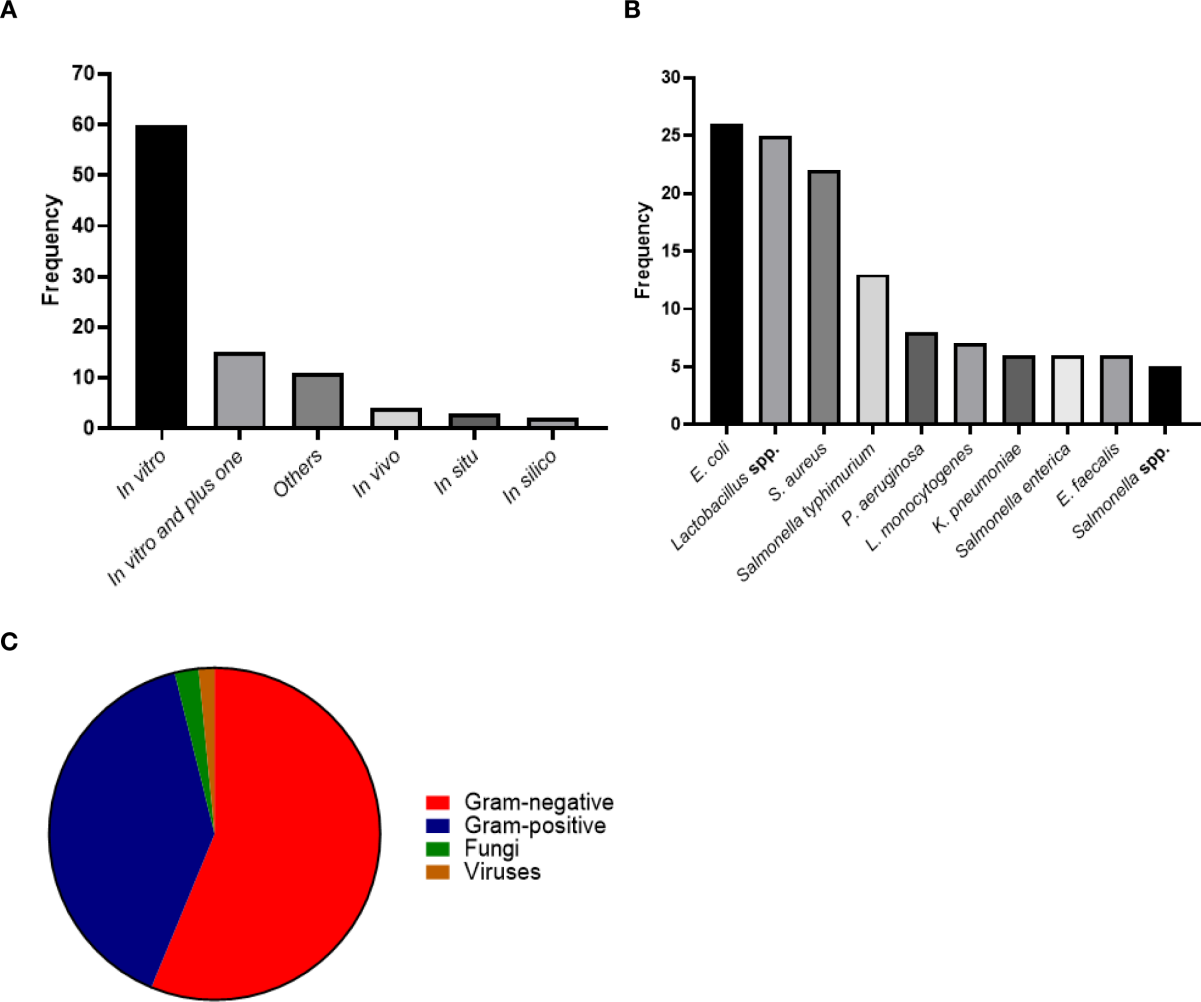
Characteristics of the included studies investigating *Limosilactobacillus fermentum* cell-free supernatants: **(A)** study designs, **(B)** targeted microorganisms, and **(C)** microbial group classification.

These findings indicate that, although most studies focus on traditional laboratory experiments, there is a growing diversity of innovative and integrative approaches, reflecting methodological advancements in research on *Lactobacillus* and its derived products (**Figure 6A**).

### 3.4 Targeted microorganisms in included studies

Analysis of the included studies revealed a wide diversity of microorganisms investigated as targets of the antimicrobial activity of cell-free supernatants. The most frequently studied pathogens were *Escherichia coli* (26 studies), a major cause of urinary tract and bloodstream infections worldwide; the producer strain itself (*Limosilactobacillus fermentum*, 25 studies), mainly explored for the characterization of its bioactive metabolites; and *Staphylococcus aureus* (22 studies), a common etiological agent of skin, soft tissue, and nosocomial infections, including methicillin-resistant strains (MRSA). Species of the genus *Salmonella* were also prominently studied, including *S. Typhimurium* (13 studies), *S. enterica* (6 studies), *Salmonella* spp. (5 studies), and other less prevalent species, totaling 26 occurrences. These pathogens are particularly relevant to foodborne disease outbreaks and public health surveillance. Other clinically and food-relevant microorganisms, such as *Pseudomonas aeruginosa* (8 studies), a leading cause of opportunistic infections in immunocompromised individuals; *Listeria monocytogenes* (7 studies), associated with severe foodborne infections; *Klebsiella pneumoniae* (6 studies), known for multidrug-resistant and carbapenemase-producing strains; and *Enterococcus faecalis* (6 studies), frequently implicated in nosocomial infections and vancomycin resistance, were also frequently investigated (**Figure 6B**).

From a *One Health* perspective, these findings highlight the broad relevance of antimicrobial activity across human, animal, and environmental health contexts, emphasizing the potential of cell-free supernatants from *L. fermentum* to contribute to integrated strategies for infection control and mitigation of antimicrobial resistance.

When classified by microbial group, Gram-negative bacteria, including *Escherichia coli*, *Salmonella* spp., *Klebsiella pneumoniae*, and *Pseudomonas* spp., were predominant, collectively representing the majority of the investigated targets. Gram-positive bacteria, such as *Staphylococcus aureus*, *Listeria* spp., *Enterococcus* spp., and *Streptococcus* spp., were also frequently studied. Fungal pathogens were less commonly reported, including *Candida albicans* and *Fusarium equiseti*, while viral targets, such as *Koi herpesvirus*, appeared even more sporadically. Studies addressing unconventional pathogens or complex microbial communities, such as the vaginal microbiota under dysbiosis, were also identified. These results underscore that cell-free supernatants have been primarily evaluated against bacterial pathogens of clinical and foodborne importance, whereas fungal and viral microorganisms remain less frequently investigated (**Figure 6C**).

### 3.5 Preparation methods and types

Analysis of the types of preparations used in the included studies revealed that the majority employed CFS alone, accounting for 42.1% of all publications. Studies utilizing mixed preparations (36.8%) were also prominent, in which the supernatant was combined with other defined fractions. Other approaches, such as the use of exopolysaccharides (EPS, 6.3%), extracts (5.3%), fermented broth (4.2%), and active cultures (4.2%), were less frequently applied, while only one study employed a purified recombinant enzyme (endolysin, 1.1%). Focusing solely on studies that incorporated some type of supernatant, pure CFS was even more predominant (66.7%), followed by EPS (10.0%) and extracts (8.3%), whereas the use of recombinant enzymes remained minimal (1.7%).

Among the evaluated preparations, CFS not only appeared most frequently but also consistently showed broad-spectrum antimicrobial and antibiofilm activity, reflecting both robust efficacy and reproducibility. In contrast, EPS and extracts, though less commonly applied, exhibited strain- or pathogen-specific effects, suggesting potential benefits that are highly context-dependent. Recombinant enzymes, while capable of potent activity, remain largely unexplored and are limited by methodological challenges, reducing their current practical applicability. Overall, this comparative perspective emphasizes that the choice of preparation can directly influence the observed antimicrobial potency, and that future studies should explore alternative strategies to uncover complementary or enhanced effects of *Limosilactobacillus fermentum*-derived bioactive compounds.

Taken together, these findings indicate that using CFS remains the most established and reliable approach for evaluating antimicrobial and antibiofilm activity, while other preparations, EPS, extracts, and recombinant enzymes, are still relatively underutilized. This gap highlights clear opportunities for methodological diversification and innovation in future research.

### 3.6 Diversity of analytical methods

Our analysis of the included studies reveals a striking methodological diversity in the investigation of *Limosilactobacillus fermentum* and its derivatives. Agar diffusion assays, the most frequently employed method (20.8%), provide initial evidence of antimicrobial activity, yet their outcomes are strongly species-dependent and do not fully capture *in vivo* relevance. Biotechnological and gastrointestinal resistance tests (18.0% and 15.2%, respectively) inform on functional resilience but fall short of quantifying antimicrobial potency directly. Molecular approaches (12.4%) uncover potential antimicrobial genes and pathways, although actual phenotypic activity can differ between pathogens. MIC determinations (9.6%) deliver quantitative and clinically relevant data, while less common assays, such as antibiofilm, enzymatic, thermal stability, and immunomodulatory tests (3–5%), offer targeted functional insights.

Specialized evaluations, including animal models, sensory and chemical profiling, physicochemical analyses, MBC, and co-culture experiments (≤1.7%), provide the most biologically and clinically meaningful evidence, though they are limited by low prevalence and complexity. Taken together, these findings suggest that the robustness and translational relevance of reported antimicrobial effects are highly dependent on both the method employed and the bacterial species studied.

Importantly, our review highlights that while classical *in vitro* approaches dominate literature, the thoughtful integration of molecular, *in vivo*, and multi-dimensional analytical strategies is critical. Only through such integrative approaches can we fully elucidate antimicrobial mechanisms and guide the development of interventions capable of mitigating microbial resistance across human, animal, and environmental contexts.

### 3.7 Microbial diversity in biofilm and antibiofilm studies

Regarding the evaluation of antibiofilm activity and the prevention of biofilm formation, nine studies were identified targeting a variety of microorganisms. The most frequently investigated pathogens were *Staphylococcus aureus* and *Escherichia coli*, each represented in four studies. Other studies included MDR strains, such as MDR *E. coli* and MDR *Enterococcus faecalis*, as well as clinically and food-relevant microorganisms, including *Streptococcus mutans*, *Listeria monocytogenes*, *Salmonella typhi*, *Vibrio parahaemolyticus*, *Candida albicans* (two studies each), *Neisseria gonorrhoeae*, *Streptococcus agalactiae*, *Klebsiella pneumoniae*, *Pseudomonas aeruginosa*, *Salmonella enterica*, and *Enterococcus faecium*, each represented by one study.

These findings highlight the diversity of microorganisms evaluated, reflecting a dual concern: addressing pathogens of both hospital and community relevance, and those associated with food contamination. Notably, the majority of studies focus on Gram-positive and Gram-negative bacteria with the highest clinical prevalence. However, more comprehensive evaluations against other MDR pathogens remain limited.

Importantly, these results underscore the critical need for strategies that not only prevent biofilm formation but also disrupt pre-established biofilms, which represent a key factor in the persistence and dissemination of MDR bacteria. Such interventions are essential for mitigating antimicrobial resistance and controlling infections across human, animal, and environmental health contexts.

### 3.8 Antimicrobial activity of *Lactobacillus* spp.

The analysis of multiple *Lactobacillus* and *Limosilactobacillus* strains demonstrated broad-spectrum antimicrobial activity against Gram-positive and Gram-negative bacteria, as well as selected fungi. High-potency strains, including *Lactobacillus* sp. T2 and *L. fermentum* SNR1, exhibited strong inhibition (+++) against key pathogens such as *S. aureus, E. coli, Salmonella*, and *Shigella*, mediated primarily by bacteriocins, antimicrobial peptides, and other bioactive metabolites. Moderate inhibition was observed in strains like *L. acidophilus* and *L. plantarum*, mainly via organic acids and hydrogen peroxide. Neutralized cell-free supernatants and bacteriocin-like inhibitory substances (BLIS) further confirmed strain-dependent antimicrobial efficacy (**Table 2**). These findings highlight the multifactorial and strain-specific nature of *Lactobacillus*-mediated inhibition and reinforce their potential application in food safety, probiotic formulations, and integrated One Health strategies to reduce pathogen prevalence and mitigate antimicrobial resistance.

**Table 2:** Antimicrobial activity of *Lactobacillus* spp. and *Limosilactobacillus* spp.

Species/strain	Tested pathogen	Observed activity	Mechanism/bioactive compound	Applied methodology	Reference
*Lactobacillus* sp. T2	*S. aureus, E. faecalis, K. pneumoniae, P. aeruginosa, E. coli, S. typhi, Shigella*	Very strong (+++)	Bacteriocins	Agar-well diffusion	Prabhurajeshwar and Chandrakanth, 2017
*Lactobacillus* sp. T4	*S. aureus, E. faecalis, K. pneumoniae, P. aeruginosa, E. coli, S. typhi, Shigella*	Strong (++)	Organic acids	Agar-well diffusion	Prabhurajeshwar and Chandrakanth, 2017
*Lactobacillus* sp. T16	*S. aureus, E. faecalis, K. pneumoniae, P. aeruginosa, E. coli, S. typhi, Shigella*	Strong (++)	Organic acids	Agar-well diffusion	Prabhurajeshwar and Chandrakanth, 2017
*L. fermentum* SNR1	*S. mutans*	Inhibition/anti-biofilm	Antimicrobial peptide (4.33 kDa)	Soft Agar-well diffusion	Repally et al., 2024
*L. fermentum* SNR1	*Listeria monocytogenes, S. aureus, V. parahaemolyticus, Salmonella Typhi*	Inhibition observed	Antimicrobial peptide (4.33 kDa)	Soft Agar-well diffusion	Repally et al., 2024
*L. fermentum* A51	*E. coli, S. aureus*	Inhibition observed	Organic acids, H_2_O_2_, bacteriocins	Agar-well diffusion	Haryani et al., 2023
*L. fermentum* SC1001	*E. coli, S. aureus, B. cereus, L. monocytogenes*	Inhibition observed	BLIS	Agar-well diffusion	Haryani et al., 2023
*Lacticaseibacillus paracasei* K2003	*E. coli, S. aureus, B. cereus, L. monocytogenes*	Inhibition observed	BLIS	Agar-well diffusion	Haryani et al., 2023
*Lacticaseibacillus rhamnosus* KF1002	*E. coli, S. aureus, B. cereus, L. monocytogenes*	Inhibition observed	BLIS	Agar-well diffusion	Haryani et al., 2023
*Lacticaseibacillus rhamnosus* MK2003	*E. coli, S. aureus, B. cereus, L. monocytogenes*	Inhibition observed	BLIS	Agar-well diffusion	Haryani et al., 2023
*L. fermentum*	*Listeria monocytogenes, E. coli, Salmonella Typhimurium, P. aeruginosa, S. aureus*	Inhibition observed	BLIS	Agar-well diffusion and microdilution methods	Ben Farhat et al., 2022
*Lacticaseibacillus paracasei*	*Listeria monocytogenes, E. coli, Salmonella Typhimurium, P. aeruginosa, S. aureus*	Inhibition observed	BLIS	Agar-well diffusion and microdilution methods	Ben Farhat et al., 2022
*Lacticaseibacillus rhamnosus*	*Listeria monocytogenes, E. coli, Salmonella Typhimurium, P. aeruginosa, S. aureus*	Inhibition observed	BLIS	Agar-well diffusion and microdilution methods	Ben Farhat et al., 2022
*L. fermentum* CCT 1629	*Salmonella* spp.	Significant inhibition	Organic acids, H_2_O_2_, bacteriocins	Minimum inhibitory concentration	Evangelista et al., 2021
*L. rhamnosus* ATCC 7469	*Salmonella* spp.	Significant inhibition	Organic acids, H_2_O_2_, bacteriocins	Minimum inhibitory concentration	Evangelista et al., 2021
*L. acidophilus* Llorente	*Salmonella* spp.	Significant inhibition	Organic acids, H_2_O_2_, bacteriocins	Minimum inhibitory concentration	Evangelista et al., 2021

Antimicrobial activity is classified as: Very strong (+++), Strong (++), Significant inhibition = quantitatively robust (e.g., MIC or log reduction), and Inhibition observed = qualitative detection of growth inhibition. BLIS: Bacteriocin-like inhibitory substances, ATCC: American Type Culture Collection, CCT: Coleção de Culturas Tropicais/Tropical Culture Collection.

## 4 Discussion

### 4.1 Overview of key findings

This review shows that CFS of *Limosilactobacillus fermentum* have been most frequently tested for antimicrobial and antibiofilm properties against both Gram-positive and Gram-negative bacteria of clinical or food relevance (Lima et al., 2007; Heredia-Castro et al., 2015; Carmo et al., 2016). Most papers used *in vitro* assays and focused on purified CFS, but the experimental designs varied widely, ranging from molecular analyses and gastrointestinal resistance tests to evaluations of antibiofilm activity and stability under enzymatic or thermal stress (Prabhurajeshwar and Chandrakanth, 2017; Ahmed et al., 2019). Several studies addressed prevention or disruption of biofilms formed by multidrug-resistant strains, which suggests that CFS may have practical value for infection control. However, most studies relied exclusively on *in vitro* designs, which limits the ability to extrapolate these findings to real infection scenarios (Das et al., 2020; De Souza Rodrigues et al., 2020; Lv et al., 2021).

### 4.2 Comparison with previous research and contextualization

While prior studies have reported antimicrobial activity of various lactic acid bacteria, our findings emphasize the unique potential of *L. fermentum* CFS in targeting pathogens across human, animal, and food contexts (Durango-Zuleta et al., 2022; Hudec et al., 2024; Repally et al., 2024; Darko and Mills-Robertson, 2025). By integrating data from diverse sources, including fermented foods, human microbiota, and environmental isolates, this review situates *L. fermentum* CFS within a One Health framework, bridging human, animal, and environmental health interventions Compared to earlier research, our synthesis underscores the broader applicability of CFS against MDR pathogens and biofilms, areas that have been less systematically addressed (Lando et al., 2023; Wei et al., 2023; Nataraj et al., 2025).

### 4.3 Implications of *Lactobacillus* spp. antimicrobial activity

Our findings show that *Lactobacillus* spp. and *Limosilactobacillus* strains show a broad range of antimicrobial actions, including against fungi. The strength and mechanism of inhibition, however, differ between strains: while isolates such as *Lactobacillus* sp. T2 or *L. fermentum* SNR1 appear to rely on bacteriocins and peptides, others act mainly through organic acids or hydrogen peroxide (Azizian et al., 2021; Repally et al., 2024; Tarannum et al., 2024; Nandi and Mandal, 2025). Recognizing this variability is key when selecting strains for specific uses. Combining probiotic LAB with broad-spectrum activity may offer a natural and sustainable way to reduce pathogen load, limit the spread of antimicrobial resistance and support human and animal health. These results support the inclusion of selected *Lactobacillus* spp. strains in food safety, probiotic development and antimicrobial stewardship initiatives (Haryani et al., 2023; Mehmood et al., 2023; Park et al., 2023; Sornsenee et al., 2024; Evangelista et al., 2025).

### 4.4 Functional and biotechnological potential of *Limosilactobacillus fermentum*

Taken together, the studies analyzed reinforce the central role of *Limosilactobacillus fermentum* and other lactic acid bacteria as microorganisms of high probiotic and biotechnological relevance (Wang et al., 2023; Lian et al., 2024; Chai et al., 2025). Regardless of their origin, from fermented foods, human breast milk, or animal microbiota, these strains exhibit a wide range of beneficial properties, including antimicrobial, antibiofilm, antioxidant, immunomodulatory, and metabolic activities (Imade et al., 2021). This diversity of effects, demonstrated through *in vitro*, *in vivo*, and omics-based approaches, highlights the potential of these microorganisms not only in promoting human and animal health but also in food preservation and quality enhancement (Imade et al., 2021; Zhang et al., 2022). Therefore, the findings converge toward the recognition of *L. fermentum* as a promising candidate for functional and therapeutic applications (Fan et al., 2024; Zhang et al., 2024; Chen et al., 2025).

### 4.5 Mechanistic insights and functional relevance

Cell-free supernatants of *L. fermentum* contain several bioactive compounds, including bacteriocins, organic acids, hydrogen peroxide and other metabolites, that contribute to growth inhibition and biofilm disruption (Javed et al., 2022; Estrada et al., 2025). Preventing the establishment of biofilms, as well as breaking down those already formed, is essential in tackling multidrug-resistant (MDR) bacteria, since biofilms provide protection and enhance tolerance to antibiotics (Ehrström et al., 2010; Adithi et al., 2024; Yuan et al., 2024; Ferdiansyah et al., 2025; Zhang et al., 2025). The reviewed studies indicate that CFS may complement conventional antimicrobials by reducing colonization and limiting the spread of pathogens in clinical, agricultural and environmental settings. Further chemical characterization will be useful to clarify the functional basis of these effects (Mishi et al., 2024; Hua et al., 2025; Xu et al., 2025).

### 4.6 Chemical and metabolic mechanisms underlying the functional properties of *Limosilactobacillus fermentum*

Chemically, the reviewed studies highlight the ability of *Limosilactobacillus fermentum* strains to produce a diverse array of bioactive metabolites, including bacteriocins, exopolysaccharides, and bioactive peptides (Ehrström et al., 2010; Adikari et al., 2021; Hossain, 2022; Marquez et al., 2022). These compounds contribute to antimicrobial, antioxidant, and immunomodulatory activities, underpinning the functional and therapeutic potential of these strains. Moreover, the metabolic versatility of *L. fermentum* is evident in its capacity to transform dietary components such as oligosaccharides, nitrites, and other substrates, further enhancing its bioactive profile (Adikari et al., 2021; Mishi et al., 2024; Hua et al., 2025). Co-culture strategies with other bacteria or yeasts have been shown to amplify the production of these metabolites, indicating synergistic chemical interactions that may optimize probiotic and functional properties. Collectively, these chemical and metabolic traits provide mechanistic insights into how *L. fermentum* exerts its beneficial effects in food systems and host health contexts (Hossain, 2022; Odumosu et al., 2023; Deng et al., 2024).

### 4.7 Probiotic and postbiotic interventions in animal models as a strategy to mitigate antimicrobial resistance

Evidence from animal models suggests that *L. fermentum* and its postbiotic derivatives can help mitigate antimicrobial resistance by shaping the gut microbiota and reducing pathogen colonization (Odumosu et al., 2023). In mice, administration of *L. fermentum* or its supernatants lowered inflammation and oxidative stress, reflecting immunomodulatory actions that strengthen host defenses (Das et al., 2020; Deng et al., 2024; Deng et al., 2024). Strains also preserved intestinal barrier function and reshaped microbial communities under *E. coli* challenge, showing the ability to compete with pathogenic and resistant bacteria. When considered alongside *vitro* antimicrobial data, these results support the use of probiotics and postbiotics as complementary tools to reduce antibiotic dependence, curb the rise of resistance and promote intestinal health (Ma et al., [[NoYear]]; Abramov et al., 2022; Gizachew et al., 2023; Fan et al., 2024). Despite promising outcomes, the number of available *in vivo* studies remains limited, and further trials in larger animals are necessary to validate these effects under practical conditions (Grishina et al., 2023; Huang et al., 2023; Nandha and Shukla, 2023; Wei et al., 2025).

### 4.8 Methodological considerations and limitations

The studies included in this review demonstrate substantial methodological heterogeneity, encompassing agar diffusion assays, minimal inhibitory concentration (MIC) determinations, enzymatic and immunomodulatory analyses, as well as diverse biotechnological and molecular approaches (Rasheed et al., 2021; Pakroo et al., 2022; Nealon et al., 2024; Odorskaya et al., 2024). While this diversity reflects the multidisciplinary interest in products derived from *Limosilactobacillus fermentum*, it significantly hampers direct comparison and reproducibility among studies. Notably, the current literature is largely dominated by *in vitro* studies, which represent 60 of the 95 studies analyzed (**Figure 6A**), requiring a more cautious interpretation when considering their translational relevance. The predominance of *in vitro* assays with well controlled environment, although providing relevant preliminary evidence, offers limited predictive value regarding *in vivo* efficacy, since does not reliably represent real-world conditions in clinical, animal, or complex environmental settings, where host–microbe interactions, immune responses, and environmental factors strongly influence the outcomes (Meng and Oh, 2021; Goa et al., 2022; Tkesheliadze et al., 2023). Moreover, variations in cell-free supernatant preparation, strain-specific characteristics, and experimental conditions further contribute to inconsistent and sometimes conflicting outcomes (Oliveira et al., 2022; Patel et al., 2023; Vilhelmova-Ilieva et al., 2023), only a limited number of investigations have employed standardized methodologies, *in vivo* validation, or integrative multi-omics approaches, which are crucial for bridging the gap between experimental findings and practical applications (Ahmed et al., 2019; Ben Farhat et al., 2022; Kumari et al., 2022; Qian et al., 2023). Therefore, future research should prioritize the development and implementation of standardized *vivo* models, alongside harmonized experimental protocols, to more accurately assess the therapeutic and translational potential of *L. fermentum*-derived postbiotics where mixed factors can influence the outcomes. Efforts like these, coupled with more in-depth mechanistic research and translational assessment, are essential to improve the reliability of the evidence and enable clinically relevant applications (Gholami et al., 2023; Rather et al., 2023; Roldán-Pérez et al., 2023).

### 4.9 Implications for one health and antimicrobial resistance mitigation

The broad-spectrum antimicrobial and antibiofilm activity of *L. fermentum* CFS underscores its potential as a One Health intervention, targeting pathogens relevant to human health, food safety, and environmental reservoirs. By preventing biofilm formation and disrupting established biofilms, CFS address a critical factor in the persistence of MDR organisms, offering an integrative strategy to mitigate antimicrobial resistance across multiple sectors (Evangelista et al., 2021; Hossain et al., 2022; Roldán-Pérez et al., 2023; Asoudeh-Fard et al., 2024; Thuy et al., 2024).

## 5 Conclusion

This systematic review brings together current evidence on the antimicrobial potential of *Lactobacillus* spp. and *Limosilactobacillus* strains, showing consistent activity against clinically relevant and foodborne pathogens. The results confirm that inhibition is strain-dependent and involves multiple mechanisms, including bacteriocins, peptides, organic acids and hydrogen peroxide. By assembling data from diverse experimental approaches, the review offers an integrated view of how probiotic lactic acid bacteria can support food safety, pathogen control and efforts to curb antimicrobial resistance. Yet, the predominance of *in vitro* evidence calls for a balanced and cautious interpretation of these findings in applied contexts. While the data clearly indicates a robust antimicrobial capacity, the translation of this potential into effective real-world applications depends on further validation in standardized *in vivo* models capable of reflecting biological and ecological complexity. Systematic reviews of this kind are essential to guide the targeted selection of probiotic strains and inform their future use in public health, food biotechnology, and antimicrobial stewardship, fostering more scientifically grounded and context-aware applications.

## Data Availability

The original contributions presented in the study are included in the article/**Supplementary Material**. Further inquiries can be directed to the corresponding author.
