# Supplementary material for: Probiotic cell-free supernatants as a strategy against antimicrobial resistance: a systematic review
Source: Front Cell Infect Microbiol. 2026 Mar 2;16:1731341. doi: 10.3389/fcimb.2026.1731341 (PMC12989548; doi:10.3389/fcimb.2026.1731341)
Supplement: Supplementary file 1 [file Table1.docx]

**SUPPLEMENTARY MATERIAL**

**Table SM1**. Overview of the Systematic Review Methodology and Data Extraction Items

| **General information** | |
| --- | --- |
| Title | **Probiotic cell-free supernatants as a strategy against antimicrobial resistance: A systematic review** |
| Researchers | Maisah Meyhr D’Carmo Sodré , Ian David Araújo Cruz, Uener Ribeiro Santos, Sheila Cristina Potente Dutra Luquetti ,Vânia Lúcia Silva , Alessandra Barbosa Ferreira Machado,Cláudio Galuppo Diniz ,Cláudio Teodoro de Souza,Carla Cristina Romano, Lauro Juliano Marin, Luciana Debortoli de Carvalho |
| Description | Cell-free supernatants (CFS), postbiotics produced by *Lactobacillus* species, represent promising natural antimicrobial agents, displaying broad-spectrum activity against pathogens, including antibacterial, antibiofilm, and antivirulence effects. They may contribute to controlling the rapid spread of antimicrobial resistance not only in healthcare settings but also in agriculture and waste management. Although several *in vitro* and *in vivo* studies have assessed the antimicrobial activity of *L. fermentum* supernatants, the evidence remains fragmented, with few comprehensive syntheses available. Understanding the scope, mechanisms, and limitations of this activity is crucial to advance the use of postbiotic products in clinical, food, and pharmaceutical applications. Therefore, this systematic review aims to evaluate the available scientific evidence on the antimicrobial potential of *Lactobacillus* spp., particularly *L. fermentum*, cell-free supernatants against pathogenic microorganisms. |
| Goal | To systematically assess the scientific evidence available in the literature regarding the antimicrobial activity of cell-free supernatants produced by *Limosilactobacillus fermentum*. |
| Specific objectives | 1. Elucidate the methods for obtaining and preparing cell-free supernatants (CFS) used in the selected studies. 2. Characterize the target microorganisms against which the *L. fermentum* supernatants were tested (Gram-positive and Gram-negative bacteria, fungi, etc.). 3. Analyze the methods used to evaluate antimicrobial activity, such as inhibition zone, MIC, MBC, among others. 4. Summarize the results found regarding the antimicrobial efficacy of L. fermentum CFS, highlighting the effects observed and their possible applications. 5. Evaluate the methodological limitations of the included studies, aiming to guide future research on the subject. |
| **Research questions** | |
| Main Question | What is the evidence on the antimicrobial activity of cell-free supernatants from *Limosilactobacillus fermentum*? |
| Population | Pathogenic microorganisms (e.g., bacteria or fungi) targeted by antimicrobial assays. |
| Intervention | Treatment or exposure to cell-free supernatants derived from *Limosilactobacillus fermentum* cultures. |
| Comparison | No treatment, untreated controls, or treatments with other antimicrobial agents or supernatants from different microorganisms (if applicable). |
| Outcomes | Antimicrobial effects such as inhibition zones, minimum inhibitory concentrations (MIC), bacteriostatic/bactericidal activity, or reduction in microbial viability and immunomodulation |
| Study | Experimental studies assessing antimicrobial activity. |
| Specific questions | What methods are employed to obtain the cell-free supernatants?  Against which microorganisms have *L. fermentum* supernatants shown significant antimicrobial activity?  What methodologies are most commonly used to assess the antimicrobial activity of the CFS?  What is the reported antimicrobial spectrum of the supernatants (e.g., Gram-positive, Gram-negative, fungi)?  Have studies identified specific bioactive components in the supernatants responsible for the antimicrobial effect?  What are the main methodological limitations and research gaps identified in the included studies? |
| Kind of study | Primary studies in the form of scientific articles that have been peer-reviewed. |
| **Identification of studies** | |
| Keywords | *Limosilactobacillus fermentum;* cell-free supernatant*;* antimicrobial activity*;* systematic review*;* postbiotics |
| Search strings | "Limosilactobacillus fermentum" OR "Lactobacillus fermentum" OR "L. fermentum"  AND "cell-free supernatant" OR "culture supernatant" OR "fermentation supernatant" OR "CFS" OR "postbiotic"  AND "antimicrobial" OR "antibacterial" OR "antimicrobial activity" OR "antimicrobial effect" OR "inhibition of pathogens" |
| Selection criteria for search sources | - Database with peer-reviewed published studies  - Available on the internet |
| List of search sources | - Science Direct  - Pubmed  -Scopus |
| Search strategy | Direct search in databases using appropriate strings |
| **Selection and evaluation of studies** | |
| Inclusion and exclusion criteria for studies | *Inclusion*:   - *In vitro, in vivo*, *in silico* experimental studies evaluating the antimicrobial activity of cell-free supernatants (CFS) obtained from *Limosilactobacillus fermentum* cultures. - Studies published in peer-reviewed scientific journals. - Articles written in English/Portuguese, - Studies reporting at least one measurable outcome (e.g., inhibition zone diameter, minimum inhibitory concentration, microbial viability reduction). - Full-text articles available for review.   *Exclusion*:   - Review articles, meta-analyses, editorials, commentaries, letters to the editor, conference abstracts, or academic theses/dissertations. - Duplicate publications or studies with insufficient or missing data for extraction and analysis. - Studies not published in English or Portuguese, or studies for which the full text was not available. |
| Strategy for initial selection of studies | Analysis of Titles and Abstracts |
| Strategy for final selection of studies | Studies that possess all the inclusion criteria and does not show any of the exclusion criteria, after the reading of the main text. |
| Assessment of the quality of studies | Failure/inconsistency between methodology and results/conclusions. |
| **Data synthesis and presentation of results** | |
| Data extraction strategy | - Author(s) and year of publication - Country of origin - Study design and methodology - Microorganism(s) tested - Strain and culture conditions of Lactobacillus sp, especially *Limosilactobacillus fermentum* - Method of cell-free supernatant (CFS) preparation - Antimicrobial assay performed (e.g., agar diffusion, broth microdilution) - Reported outcomes (e.g., inhibition zone size, MIC values, bactericidal effect) - Main findings and conclusions |
| Data summarization strategy | Tables and graphs. |
| Publishing strategy | Scientific journal of relevant impact in the area. |
